# Effects of High-Intensity Ultrasound Treatments on the Physicochemical and Structural Characteristics of Sodium Caseinate (SC) and the Stability of SC-Coated Oil-in-Water (O/W) Emulsions

**DOI:** 10.3390/foods11182817

**Published:** 2022-09-13

**Authors:** Xiangli He, Shangxi Jia, Jiayun Wan, Yan Li, Yanyan Zhang, He Zhu, Ke Li

**Affiliations:** 1 Henan Key Laboratory of Cold Chain Food Quality and Safety Control, College of Food and Bioengineering, Zhengzhou University of Light Industry, Zhengzhou 450001, China; 2 School of Food Science and Technology, Shandong Agriculture and Engineering University, Jinan 250100, China

**Keywords:** high-intensity ultrasound, sodium caseinate, physicochemical, structure, emulsion stability

## Abstract

The effects of high-intensity ultrasound treatment (0, 3, 6, 9 min) on physicochemical and structural characteristics of SC and the storage, thermal and freeze–thaw stability of SC O/W emulsions were investigated. The results showed that ultrasound treatment reduced the particle size of SC, although there were no obvious changes in zeta potential, profiles and weights. Ultrasound treatment improved surface hydrophobicity and fluorescence intensity of SC and changed ultraviolet–visible (UV–Vis) spectroscopy but had no influence on the secondary structure of SC. This indicates that ultrasounds might destroy the tertiary structure but leave most of the integral secondary structure. A scanning electron microscope (SEM) also showed that ultrasound-treated SC presented small aggregates and a loose structure. The physicochemical and structural changes of SC benefited the ability of protein adsorbing oil droplets and emulsion stability. Under stresses such as storage, thermal and freeze–thawing, the oil droplets of treated emulsions were still uniform and stable, especially at 6 min and 9 min. Overall, the high-intensity ultrasounds made the SC present small aggregates and a loose structure improving the SC O/W emulsions stability under storage, thermal and freeze–thawing environment and have great potential to stabilize the SC prepared O/W emulsions.

## 1. Introduction

O/W emulsions and water-in-oil (W/O) emulsions are two common types of emulsions. O/W emulsions are more popular than the latter because of their compatibility with water and their convenience when cleaning [1]. O/W emulsions are comprised of oil as a dispersed phase and liquid as a continuous phase and have been extensively used in emulsion-type sausages, mayonnaise, beverages and dressings in the food industry [2]. O/W emulsions are prone to phase separation, flocculation, aggregation, coalescence and Ostwald ripening due to their thermodynamic instability, which affects the texture and sensory and nutritional properties of emulsion-based food [3,4]. However, for practical applications, there needs to be a relatively long time for emulsions to remain stable. Moreover, thermal treatments and the freeze-thawing process may occur in emulsion-based food products [5]. Therefore, it is necessary to enhance the stability of O/W emulsions. Fortunately, interface engineering is a promising approach that can enhance the stability of O/W emulsions, especially the thickness and structure of the interface. Acidified casein (SC) is one of the most commercially valuable proteins in food processing, consisting of four phosphoproteins: α_s1_-, α_s2_-, β- and k-casein [6]. The surface activity of SC mainly depends on α_s1_- and β-casein. There are hydrophobic regions at the ends and in the middle of α_s1_-casein, while β-casein has hydrophilic and hydrophobic regions [7]. SC can quickly adsorb to the O/W interface because of its hydrophobic and hydrophilic regions with fine emulsifying properties [8]. These characteristics, and the fact that it is a natural component, make SC a general emulsifier in food emulsions.

Some studies have reported that high-intensity ultrasounds can influence the physicochemical and structural characteristics of casein and its derivatives. De Figueiredo Furtado et al. reported that SC treated by ultrasound (at 300 W and 20 kHz for 0, 2, 4 or 6 min) had a small particle size and high surface hydrophobicity, but no change was observed in the secondary structure of the proteins [9]. Bi et al. also demonstrated that the chemical structure of ultrasound-treated casein was not influenced by a Fourier transform infrared spectroscopy (FTIR) analysis [10]. However, the study presented an increase in the anti-parallel structure and β-turn structure of casein by ultrasonic treatment (0, 100, 200, 300 and 400 W at 10 min) and a slight effect in solubility [11]. Zhang et al. found that a high-intensity ultrasound (20 kHz, 58 W/L at 0, 0.5, 1, 2 and 5 min) had a positive influence on the solubility, emulsification and surface hydrophobicity of micellar casein concentrates [12]. Moreover, the ultrasound increased β-sheets and random coils while decreasing α-helix and β-turn contents, illustrating the secondary structural changes of proteins. The physicochemical and structural changes of ultrasound-treated SC were debatable; this may be due to ultrasonic conditions, protein concentration, sample volume and solvents. 

In our previous study, we successfully applied a high-intensity ultrasound to enhance the thickness of the interface of SC-coated O/W emulsions and improve the stability of emulsions [13]. However, proteins as emulsifiers—the parent physicochemical and structural changes of protein before its adsorption to the O/W interface—play a significant role in the stability of final emulsions [14], such as solubility, particle size and surface hydrophobicity. We have not paid too much attention to the relation between the stability of SC-coated O/W emulsions and the physicochemical and structural characteristics of SC by high-intensity ultrasounds. According to our knowledge, few investigations have explored changes in the physicochemical and structural characteristics of SC under our experimental conditions. Meanwhile, the application of ultrasound on the pre-emulsification of plant lipids improved the functional properties of myofibrillar protein–soybean oil composite gel. However, the analysis of thermal and freeze–thaw stability of pre-emulsion was rarely explored. During industrial meat processing, the storage and freeze–thaw stability are related to the long term storage of pre-emulsion. The thermal stability of pre-emulsion is related to the cooking quality of meat products. Thus, it is necessary to explore what will happen to proteins and understand the effect of these changes on emulsion stability.

The main purpose of this study was to explore the effects of physicochemical and structural characteristics of SC on the stability of SC-coated O/W emulsions through a high-intensity ultrasound treatment. We determined the particle size and zeta potential of SC. SDS-PAGE, FTIR, UV–Vis spectroscopy, intrinsic fluorescence and SEM were further analyzed. Meanwhile, the emulsifying properties, zeta potential, storage, and thermal and freeze–thaw stability of ultrasound-treated emulsions were also determined. The proposed mechanism in terms of the relation between the physicochemical and structural characteristics of SC and their emulsion stability is discussed at the end of this article.

## 2. Materials and Methods

### 2.1. Materials

SC (Sigma-Aldrich, Shanghai, China) was used in this experiment. Soybean oil was purchased from Yihai Kerry Co., Ltd. (Zhengzhou, China). All other reagents were of analytical grade or above. Deionized water was used in the total experiments.

### 2.2. Preparing O/W Emulsions and the High-Intensity Ultrasound

The preparation of O/W emulsions and the high-intensity ultrasound was based on our previous research [13]. Succinctly, the final emulsions contained 45 wt% (*w*/*w*) continuous phase (44 wt% phosphate buffer (0.6 M NaCl, 50 mM Na_2_HPO_4_/NaH_2_PO_4_, pH 6.25), 1 wt% SC) and 55 wt% dispersed phase (soybean oil). The two phases (150 g) were mixed and homogenized by Ultra-Turrax (Angni Co., Ltd., Shanghai, China) at 10,000 rpm for 1 min and then finally, the high-intensity ultrasound-treated emulsions for 0, 3, 6 or 9 min. The ultrasound frequency was 20 kHz, and the ultrasound intensity was 50.42 ± 3.13 W/cm^2^. The emulsions were used for further analysis. The emulsions were stored at 4 ℃ for the determination of storage ability.

### 2.3. Physicochemical and Structural Analysis 

To further study the effect of the high-intensity ultrasound treatment on SC in the emulsion system, the particle size, zeta potential, SDS-PAGE, FTIR, UV–Vis spectroscopy, intrinsic fluorescence, surface hydrophobicity and SEM of ultrasound-treated SC were evaluated. The samples were prepared following the steps detailed in Section 2.2 without adding soybean oil.

#### 2.3.1. Particle Size and Zeta Potential Measurement

The average particle size and zeta potential of the samples were determined through the Zetasizer Nano-ZS 90 (Malvern Instruments, Malvern, UK) according to Tabilo-Munizaga et al. [15] and Li et al. [2] with minor changes. All the samples were diluted 100-fold with the phosphate buffer (50 mM Na_2_HPO_4_/NaH_2_PO_4_, pH 6.25). Sample particle sizes and zeta potential were then evaluated.

#### 2.3.2. SDS-PAGE

The protein composition of samples was analyzed by SDS-PAGE under reducing conditions. The protein concentration of the sample was fixed at 0.4 wt%. A 12% acrylamide separating gel and a 5% acrylamide stacking gel was used to perform the SDS-PAGE analysis as described by Laemmli [16] and Li et al. [17].

#### 2.3.3. FTIR

The samples were lyophilized for 24 h by the Lab-1-50 lyophilizer (Boyikang Experimental Instrument Co., Ltd., Beijing, China). We mixed 1 mg of lyophilized powder with 150 mg KBr for the FTIR analysis. Then, spectrums were collected by an infrared spectrometer (Vertex 70, Bruker, Germany) at a resolution of 4 cm^−1^ per point and 4000–400 cm^−1^ were recorded with 64 time scans. The amide I band of 1700–1600 cm^−1^ was analyzed using Peak Fit software (version 4.12, SPSS Inc., Chicago, IL, USA) to obtain data on secondary structure [18].

#### 2.3.4. UV–Vis Spectroscopy

The UV–Vis spectrums of samples were conducted by a spectrophotometer (TU-1810, General Instrument Co., Ltd., Beijing, China) according to the method of Li et al. [18], with slight modifications. The samples were diluted to 0.01 wt% in the phosphate buffer (50 mM Na_2_HPO_4_/NaH_2_PO_4_, pH 6.25) and were recorded as ranging from 220 to 500 nm. The second-derivative spectrum (d^2^A/dλ^2^) of the UV–Vis spectrum was calculated using Origin 8.5.

#### 2.3.5. Intrinsic Fluorescence Spectroscopy

The intrinsic fluorescence spectrum was performed by the fluorescence spectrophotometer (F-7000, Hitachi Corp., Tokyo, Japan) equipped with the 1 cm path length cell. The samples were dissolved in the phosphate buffer (50 mM Na_2_HPO_4_/NaH_2_PO_4_, pH 6.25) with a concentration of 0.01 wt%. The excitation wavelength was 280 nm (slit = 2.5 nm), the emission wavelength range was 290–450 nm, and the scanning speed was 40 nm/s. 

#### 2.3.6. Surface Hydrophobicity

Protein surface hydrophobicity was conducted according to de Figueiredo Furtado et al. [9] with minor modifications. In this study, 1-Anilino-8-naphathalene-sulfonate (ANS) was used as the fluorescent probe. The protein concentration was diluted to range from 0.0125 to 0.075 wt%. The 4 mL diluted proteins were mixed with 20 μL ANS solution (8 mM). The mixture was incubated in a dark place for approximately 10 min. The relative fluorescence intensity (RFI) was measured. The excitation and emission wavelengths were set at 390 nm and 470 nm (slit = 2.5 nm). S_0_-ANS was analyzed from the initial slope of the linear regression of the plot of RFI against protein concentration (wt%).

#### 2.3.7. SEM

The microstructure of samples was recorded by SEM (JSM-6490LV, JEOL, Japan). The samples were scattered on a two-side conductive adhesive, with gold (10 nm) coating used for the SEM observations (electron mode, 20 kV).

### 2.4. Emulsifying Properties

The emulsifying properties of ultrasound-treated emulsions were conducted according to the methods of Sui et al. [19] and Shi et al. [20] with slight modifications. After the ultrasound treatment, 20 μL of the emulsion was immediately added to the 0.1% (*w*/*v*) sodium dodecyl sulfate (SDS) solutions (5 mL), and absorbance was determined at 500 nm (A_0_). After 10 min, the same operation occurred, and the emulsion (20 μL) was mixed with the SDS solutions, and its absorbance was also measured at 500 nm (A_10_). These were calculated according to Equations (1) and (2) for the emulsifying activity index (EAI) and emulsion stability index (ESI), respectively.

(1) $${\mathrm{EAI}(m}^{2}/g)=\frac{2\times 2{.303\times A}_{0}D}{{(1-\varphi )\times C\times 10}^{4}}$$

(2) $$\mathrm{ESI}\left(\%\right)=\frac{A_{10}}{A_{0}}\times 100\%$$

Here, A_0_ and A_10_ are the absorbances of emulsions measured at 0 min and 10 min, D is the dilution factor, φ is the proportion of the oil phase, and C is the concentration of protein (g/mL).

### 2.5. Zeta Potential

The zeta potential of emulsions was performed according to the process set out in Section 2.3.1.

### 2.6. Influence of Environmental Stresses on Emulsions

We investigated the influence of the storage, thermal and freeze–thaw stabilities of emulsions, prepared as described above.

#### 2.6.1. Storage Stability 

The average particle size of prepared SC-coated emulsions during storage at 0, 7 and 14 days was determined. The determination method was based on Section 2.3.1. The microstructure of emulsions at 0 and 14 days was observed through optical microscopy. The diluted emulsions (about 5 μL) were dripped in the center of a glass slide and slowly covered the cover glass to ensure that no bubbles were formed. The microstructure of emulsions was obtained through the microscope (PH50-2A43L-PL, Phenix Optics Co., Ltd., Guangzhou, China), and the image was observed under a 100× objective lens.

#### 2.6.2. Thermal Stability

The thermal stability of emulsions by ultrasound treatment was evaluated. The emulsions (10 mL) were placed in a capped glass tube and heated in a water bath at 90 °C for 30 min. The emulsions were cooled at room temperature. The particle size distribution, visible appearance and microstructure of the emulsion were observed.

#### 2.6.3. Freeze–Thaw Stability 

The freeze–thaw stability of high-intensity ultrasound-stabilized emulsions was determined according to the methods of Taha et al. [21] with proper modifications. The emulsions (10 mL) were placed in a capped glass tube. These tubes were stored in the −20 °C refrigerator for 48 h and then thawed at room temperature. The particle size distribution, visible appearance and microstructure of the emulsion were observed.

### 2.7. Statistical Analysis

Results were presented as the means of three determinations. Duncan’s test (*p* < 0.05) was applied by SPSS 21.0. The independent experiments were performed in triplicates on different occasions. 

## 3. Results and Discussion

### 3.1. Physicochemical Properties and Structure

#### 3.1.1. Particle Size and Zeta Potential

The effect of the high-intensity ultrasound treatment on the particle size and zeta potential of SC was investigated. SC solutions at 1 wt% concentration were treated for 0, 3, 6 and 9 min; the particle size and zeta potential of SC are shown in Table 1. The results show that the particle size of SC solutions was significantly reduced (*p* < 0.05) for the ultrasound time from 0 to 6 min. There was no prominent decrease in the particle size of SC solutions for the ultrasound treatment from 6 to 9 min (*p* > 0.05). The granules of SC solutions were dispersed into smaller sizes as a consequence of the cavitation effect of the ultrasound [9]. Parallel to our findings, the particle sizes of a series of proteins, such as soy protein isolate [22], album protein isolate [23], actomyosin [24] and milk protein concentrate [25], were reduced after the ultrasound treatment. A reduction in particle size can enhance the functional properties of proteins, such as emulsifying properties [26]. In addition, the absolute values of zeta potential did not vary significantly (*p* > 0.05) after the ultrasound treatment. It may be that the ultrasound treatment conditions in our study have relatively little influence on the chemical characteristics of SC solutions [27]. De Figueiredo Furtado et al. [9] also reported that the zeta potential value of SC solutions treated by ultrasound (20 kHz, 300 W) did not change with the increase in ultrasound time.

#### 3.1.2. SDS-PAGE

**Figure 1:**
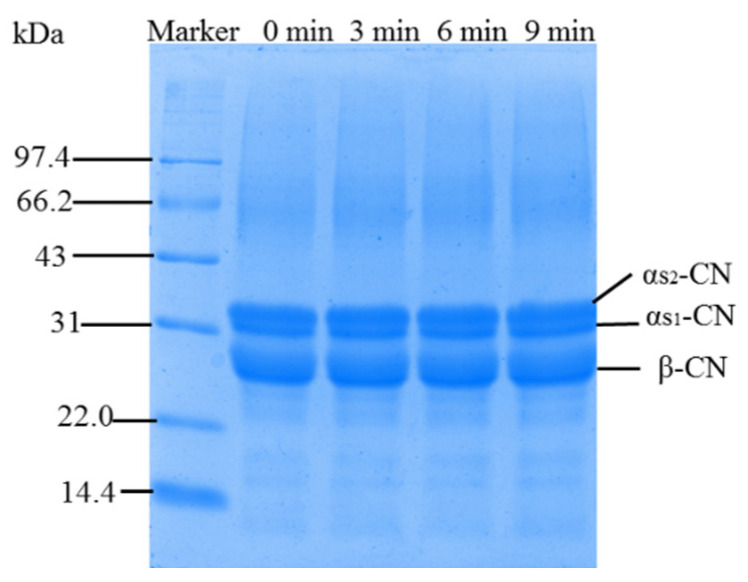
SDS-PAGE pattern of high-intensity ultrasound treated SC.

The effects of increased surface hydrophobicity, and reduction of particle size on the interaction between protein molecules and the formation of dissociation, were analyzed by the SDS-PAGE. There was no difference in SC profiles and weights before and after the ultrasound treatment (see Figure 1). The results show that the ultrasound did not change the primary structure of the SC. It can be reasonably speculated that the polypeptide backbone of SC is possibly invalidly broken up for the cavitation effect generated by the ultrasound treatment, while ionic strength or interactions of the hydrogen bond between proteins may be easily changed [28]. Saleem et al. investigated how low-frequency sonication under experimental conditions (frequency: 20 kHz, power: 120 W, time 0, 5, 10, 20 and 30 min) did not lead to any fragmentation of primary myofibrillar protein [29]. Wang et al. also found no significant difference in bands between chickpea protein isolate with and without ultrasound treatment (20 kHz at 300 W for 0–20 min) in non-reduced and reduced conditions [1]. Furthermore, inverse views observed that the molecular weight of WPI and WPC treated by ultrasound (20 kHz, ~48 W cm^−2^ and 15 min) were decreased [30]. The above phenomena may be caused by distinct ultrasound conditions, sample volumes, protein types and other factors.

#### 3.1.3. FTIR

**Figure 2:**
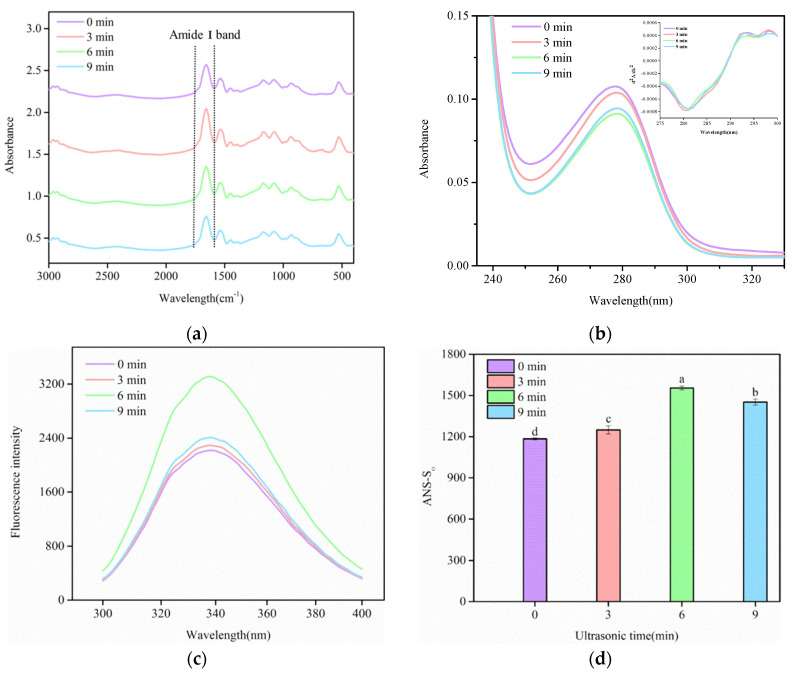
Changes in the structure of SC as a result of high-intensity ultrasound treatment. (**a**) FTIR spectrum; (**b**) UV–Vis spectroscopy; (**c**) intrinsic fluorescence spectroscopy; (**d**) surface hydrophobicity. Different letters above standard deviation bars are significantly different (*p* < 0.05).

Secondary structure was determined by FTIR, which is a common technique. Three important regions indicate changes in the secondary structure of proteins. These may be observed from the FTIR spectrum: amide I band 1700–1600 cm^−1^, amide II band 1600–1500 cm^−1^ and amide III band 1500–1100 cm^−1^ [31]. The most useful for observing the α-helix, β-turn, β-sheet and random coil is the amide I band. The region is primarily in contact with the in-plane bending of N-H groups in peptides, Cα-C-N bending and the stretching vibration of C=O groups, on some level, including the stretching vibration of C-N in peptide bonds [32]. Figure 2a shows the effect of different ultrasound times on the FTIR spectrum of SC. The peak positions of the amide I band did not change at 1656.78 cm^−1^ before and after the ultrasound. The spectrum roughly reflected secondary structural changes in the protein. The specific data of the secondary structure is shown in Table 2. The α-helix, β-sheet, β-turn and random coil had no significant difference (*p* > 0.05) for different ultrasound times. The results indicate that the secondary structure of SC was altered by the ultrasound treatment. Cavitation generated by the ultrasound might destroy the tertiary structure but keep most integral secondary structural fragments [33]. There are some studies that have reported that the primary structure of proteins was not changed by high-intensity ultrasounds [9,10,34]. Nevertheless, Yang et al. demonstrated that α-helix and β-turn were slightly decreased by ultrasounds in rice protein, while β-sheet and random coil were increased [35]. Ma et al. (2018) indicated that α-helix and β-sheet were increased and that the random coil of β-lactoglobulin was decreased after ultrasound treatments [26]. These distinct results may be due to protein features [34].

#### 3.1.4. UV–Vis Spectroscopy

The tertiary structure of proteins was obtained from the UV–Vis spectrum. Aromatic amino acid residues, including tyrosine (Tyr), tryptophan (Trp) and phenylalanine (Phe), are sensitive to the environment. Therefore, structural alternations of proteins can be obtained from the absorption spectrum and maximum wavelength [36]. Figure 2b shows the zero-order and second-derivative spectrum of SC by ultrasound treatment. The absorption peak at about 278 nm manifested the presence of aromatic amino acid residues in SC, and the zero-order spectrum slightly shifted toward a longer wavelength after the ultrasound treatment, which indicates the structural change of SC. Compared with the zero-order UV–Vis spectrum, the second derivative of the UV–Vis spectrum handles overlapping bands in the zero-order spectrum and the independent effects of aromatic side chains, indicating changes to aromatic amino residues in the microenvironment [37]. These were present in the second-derivative spectrum that had two peaks of ~292 nm and ~297 nm and two troughs of ~281 nm and ~295 nm. The peak at 292 nm is attributed to the synergic contributions of Tyr and Trp residues, indicating fluctuations in the amount and distribution of both residues. The peak for Tyr and Trp appeared as a blue shift by ultrasound, and the blue shifts of the second-derivative spectrum bands generally implied increased solvent polarity. The results show that the ultrasound treatment caused the tyrosine and tryptophan, which were exposed to the surface of the protein due to conformational changes, and the movement of Tyr and Trp to a high environmental polarity [38]. 

#### 3.1.5. Intrinsic Fluorescence

The exposure magnitude index of aromatic amino acid residues is generally measured by intrinsic fluorescence, providing further information about the structural changes of proteins. The location of Trp in proteins, in particular, makes a significant contribution to intrinsic fluorescence. Furthermore, Trp is particularly sensitive to the polarity of the microenvironment for monitoring tertiary structural changes in proteins [39]. As shown in Figure 2c, the wavelength was around 338 nm, which is where the maximum fluorescence emission occurred. It was attributed to the exposure of the previously buried Trp and a hydrophobic portion of protein, and fluorescence intensity was gradually increased by the ultrasound [40]. Vera et al. reported that increased fluorescence intensity of quinoa proteins might be due to high-intensity ultrasound changes in protein structure and conformation [41]. In addition, there was a fluorescence quenching phenomenon in the ultrasound time from 6 to 9 min. Fluorescence quenching may be longer, as the ultrasound time buried some Trp by inducing the hydrophobic rearrangement of SC. These results indicate that the ultrasound treatment influenced the weak force of hydrophobic interaction and the tertiary structure of SC.

#### 3.1.6. Surface Hydrophobicity

Surface hydrophobicity is related to the functional properties of proteins, indicating the number of hydrophobic groups on the surface of protein molecules, which can be a factor in the structural change of proteins. ANS, an effective fluorescence probe, was used due to its high specificity to hydrophobic sites. The change of S_0_-ANS indicated structural alternation and protein denaturation in proteins. The S_0_-ANS of SC, with the effect of ultrasound time, is shown in Figure 2d. The S_0_-ANS of proteins increased for up to 6 min of the ultrasound. This may be due to the effects of the cavitation phenomenon, which reduced particle size, induced a certain unfolding of the proteins and some new surface area, and caused exposure in the number of hydrophobic groups to the surrounding polar environment [23,42]. Increased surface hydrophobicity could improve the flexibility of SC, thereby improving the protein’s potential to absorb at the interface of oil droplets [33]. However, S_0_-ANS was decreased after the prolonged ultrasound, illustrating that a longer ultrasound time might lead to partial denaturation of proteins. The partially denatured proteins might cause more extensive bonding, reducing surface hydrophobicities [43]. A similar effect was observed when an egg white was treated with ultrasound [44]; it was possible that excessive ultrasound treatment made protein monomers form dimers or polymers. In addition, the variations of S_0_-ANS of treated SC exactly matched the varying tendency of fluorescence intensity.

#### 3.1.7. SEM

**Figure 3:**
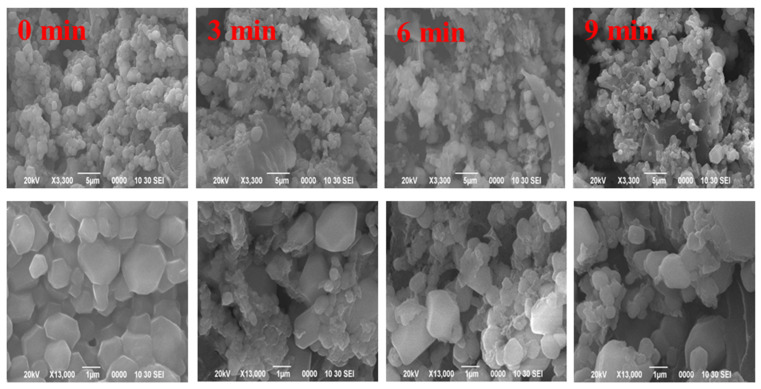
Microscopic images (SEM) of high-intensity ultrasound treated SC.

SEM was conducted to observe whether the ultrasound treatment could alter the morphology of SC. The microstructure of ultrasound-treated SC is shown in Figure 3 at two magnifications (3300× and 13,000×). SC particles are roughly spherical with heterogeneous diameters. The native SC presented aggregates and a compact structure. After the ultrasound treatment, the big aggregates were broken into small fragments and formed a loose structure. This result was similar to casein [45] and sunflower protein isolates [46]. This may be due to the fact that the cavitation effect can temporarily disperse aggregates and break covalent bonds in polymer chains, causing depolymerization of macromolecules. This result may increase molecule flexibility and make SC absorb quickly in the O/W interface, forming a dense interface film during the emulsification process and improving the stability of emulsions.

### 3.2. EAI and ESI

**Figure 4:**
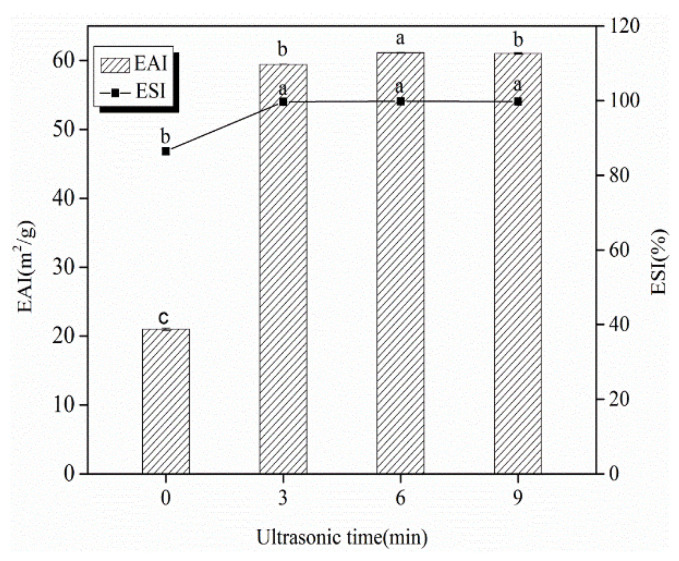
Changes in emulsifying properties of SC-coated O/W emulsions as a result of the high-intensity ultrasound treatment. Different letters above standard deviation bars are significantly different (*p* < 0.05).

The EAI and ESI generally evaluated the emulsifying properties of proteins. The EAI expresses the interfacial area stabilized per unit weight of a protein, characterizing the ability of proteins to be absorbed in the O/W interface. Meanwhile, the ESI indicates the ability of proteins to maintain emulsion stability [47]. As shown in Figure 4, the EAI significantly increased from 21.00 m^2^/g to 61.13 m^2^/g in the first 6 min ultrasound treatment (*p* < 0.05). No significant difference with the longer treatment (*p* > 0.05) was observed. The improvement illustrated that the ultrasound enhanced emulsion formation. Moreover, the ESI of SC was also higher in all the treated emulsions than in untreated emulsions, indicating that the ultrasound enhanced emulsion stability, and the emulsions did not result in phase inversion, flocculation, aggregation, coalescence and Ostwald ripening. Similarly, ultrasounds enhancing the emulsifying properties of different types of proteins have been reported by other researchers [12,18,40,48]. The enhancement in the emulsifying properties of proteins upon ultrasound was related to particle size reduction and the increase of surface hydrophobicity [49]. The decreased particle size offered an increased surface area of proteins and was quickly adsorbed to the O/W interface, which reduced interfacial tension. While ultrasounds promote the partial unfolding of proteins, causing an increase in surface hydrophobicity, enhancing hydrophobic groups exposes them to interaction with oil droplets.

### 3.3. Zeta Potential of Emulsions

**Figure 5:**
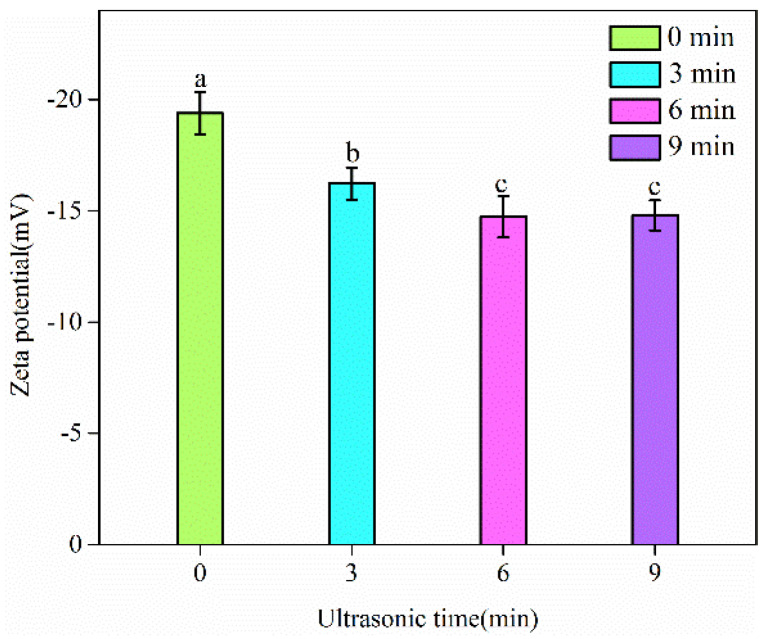
The zeta potential of oil droplets in SC stabilized emulsions. Different letters above standard deviation bars are significantly different (*p* < 0.05).

The zeta potential of SC-coated emulsions as a function of ultrasound time (0–9 min) is presented in Figure 5. The zeta potential of untreated emulsion droplets was negative (−19.38 mV). Zhao et al. also reported that the zeta potential of the emulsions (10% corn oil and 1% SC) of oil droplets was negative (−23.15 mV) [6]. This result occurred because the SC molecule presents the negative charge at a pH value higher than the isoelectric point (4.6–5.5) [27]. The ultrasound caused a significant decrease in the surface charge of SC-stabilized emulsions (*p* < 0.05), suggesting that the ultrasound decreased repulsion forces among emulsion droplets. It is noteworthy that the zeta potential of SC-coated droplets showed no significant difference between 6 and 9 min of the ultrasound treatment (*p* > 0.05). The surface charge reduction of the ultrasound treatment was attributable to the increased EAI of the emulsion (Figure 4). Sui et al. found that the decreased absolute zeta potential value was induced by protein aggregates [19]. More adsorbed proteins aggregated in the O/W interface could reduce the surface charge of oil droplets [21]. The change in the absolute zeta potential value of the emulsion droplets was also related to the native structure of biopolymers induced by the cavitation effect [21,50]. In addition, Silva et al. found that although the zeta potential of biopolymers was significantly influenced by ultrasounds, electrostatic stabilization was not the dominating mechanism of annatto seed oil emulsions [50]. The particles had a low density of surface charge (below −30 mV), which electrostatically contributed to stabilizing the emulsions [50,51,52]. The stabilization mechanism for ultrasound-induced emulsions prepared by SC as an emulsifier needs further study.

### 3.4. Impact of Environmental Stresses on Emulsion Stability

#### 3.4.1. Storage Stability

**Figure 6:**
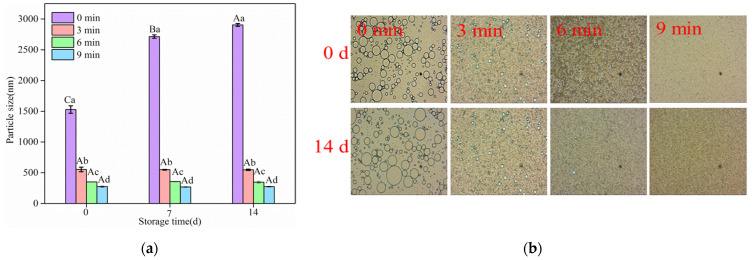
Changes in the particle size and microstructure of SC-coated O/W emulsions at different storage times. (**a**) Particle size; (**b**) microstructure; a–d: different letters indicate significant differences among the means within the same storage time (*p* < 0.05). A–C: different letters indicate significant differences among the means within the same ultrasound time (*p* < 0.05).

As shown in Figure 6a, ultrasound treatment significantly decreased the oil droplet size of emulsions as time increased (*p* < 0.05). Ultrasounds treated for 3 min decreased the initial oil droplet size from 1529.3 nm to 553.1 nm. When the time-dependent reduction effect of the ultrasound was exhibited at 6 min and 9 min, it produced emulsions with a mean size of 351.9 nm and 274.6 nm, respectively. Furthermore, the droplet size of the untreated emulsion was found to have increased to 2903.5 nm after 14 days of storage. However, for all the ultrasound-treated emulsions stored for 14 days, the average oil droplet size of the emulsions exhibited a trend of no significant increase (*p* > 0.05). The results confirmed that the ultrasound decreased emulsion droplets and prevented the aggregation behavior of emulsion oil droplets, enhancing emulsion stability. Li and Xiang [53] reported that a high-intensity ultrasound effectively decreased the average oil droplet size of 5% coconut oil O/W emulsions and created a narrow droplet size distribution range. A high-intensity ultrasound treatment formed more stable emulsions stabilized by biopolymers, showing a narrow droplet size distribution with a smaller particle size [3]. The cavitation effect could decrease oil droplets into a smaller particle size, thus producing a more stable emulsion [54,55].

Microphotographs visually observed the microstructure of emulsions during storage at a magnification of 100×. As shown in Figure 6b, microphotographs were consistent with this phenomenon in particle size. Compared with ultrasound treatments, at 0 days, the oil droplets of untreated emulsions had the largest sizes and heterogeneous distribution, which resulted in aggregation in the continuous phase. When storage time reached 14 days, the size of the oil droplets continued to increase, which may have been smaller oil droplets merging into larger ones. The larger sizes accelerated the coalescence, creaming and flocculation of emulsions because the speed of the moving droplet is proportional to the square of its radius [56] and causes instability of the emulsions. The oil droplets presented in treated emulsions gradually decreased and had a regular distribution from 3 min and 9 min, and the microstructures retained were constant with a prolonged storage time. The aggregation of oil droplets could be inhibited by reason of the cohesive interfacial films formed by SC adsorbing on the surfaces of droplets [2]. These results indicate that the ultrasound was conducive to emulsion stability. Lad et al. [57] and Kaltsa et al. [58] also investigated how oil-in-water emulsions from ultrasounds produced small droplet sizes, which were stable during the whole storage period.

#### 3.4.2. Thermal Stability

**Figure 7:**
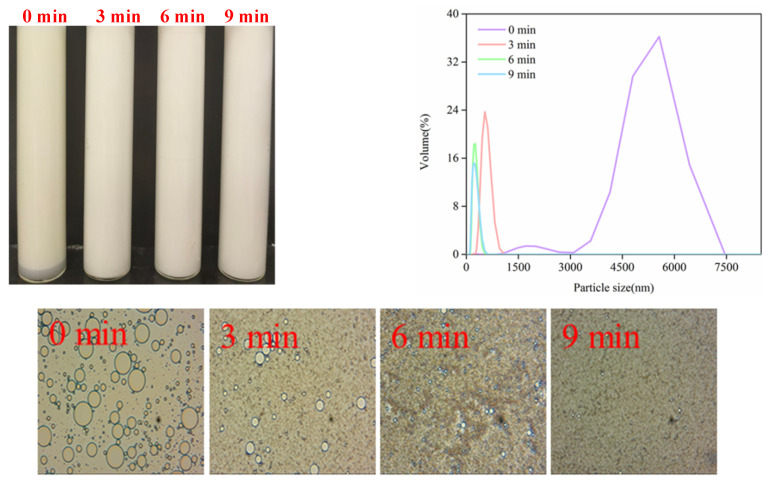
Effect of thermal treatment on the visible appearance, particle size distribution and microstructure of SC-coated O/W emulsions by a high-intensity ultrasound treatment.

High-intensity ultrasound treatment emulsions were placed in water baths at 90 °C for 30 min and naturally cooled to room temperature. The visible appearance, particle size distribution and microstructure of emulsions are presented in Figure 7. The emulsions’ thermal stability was dependent on ultrasound time. No obvious evidence of phase separation was observed, except for the unheated emulsions at 0 min. Compared with the unheated emulsions (Figure 6a), the range of particle size distribution increased at ultrasound times of 0 min and 3 min. Moreover, there was a larger size of oil droplets observed from the microstructure. Two groups of oil droplets probably occurred due to aggregation and coalescence after heat treatment. When the ultrasound time was greater than 3 min, we observed no significant changes in the visible appearance, particle size distribution and microstructure of emulsions. The emulsions were stable against heat treatment. The particle size of fresh emulsions contributed to the above results. At the ultrasound times of 6 min and 9 min, the emulsions’ particle size could be divided into nanoemulsions in the range of 20–500 nm [1]. Nanoemulsions have comparatively physical stability, suppressing droplet aggregation, coalescence and gravitational separation due to their fairly minor particle size [59]. Jin et al. [60] also reported that there was no significant change (*p* > 0.05) in the mean particle size of the fabrication of β-conglycinin, β-conglycinin/sodium dodecyl sulfate and β-conglycinin/polyethylene glycol 10,000 stabilized nanoemulsions, which were sufficiently stable against oil droplet aggregation at 80 °C for 15 min. In addition, studies have reported a large particle of WPI-stabilized β-carotene nanoemulsions, which occurred after extensive aggregation at 60 °C for 4 h [61]. The differing results may depend on heating time. Compared with shorter heating, oil droplet aggregation will appear as a result of longer heat-enhancing protein denaturation and protein–protein interactions [62]. 

#### 3.4.3. Freeze–Thaw Stability

**Figure 8:**
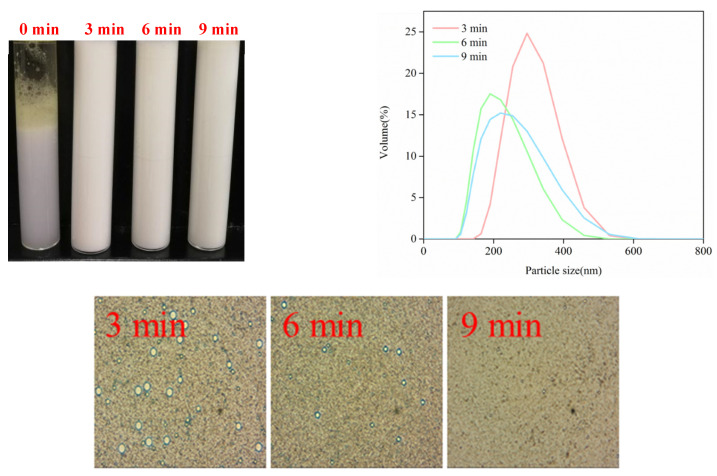
Effect of freeze–thaw treatment on the visible appearance, particle size distribution and microstructure of SC-coated O/W emulsions by a high-intensity ultrasound treatment.

Freeze–thaw stability is an important property for many emulsion-based foods, which pass through a freeze–thawing process prior to consumption. Therefore, we explored the freeze–thaw stability of high-intensity ultrasound treatment emulsions. As shown in Figure 8, it can be observed that emulsions without an ultrasound treatment were destabilized and presented great oiling off, coalescence and creaming after the freeze–thawing process. However, Zhu et al. [63] reported that Na-CN-stabilized emulsions were stable against oiling off even through three cycles of freeze–thawing treatments. These distinct results possibly depend on different dispersion ratios, protein concentrations and emulsification methods in the emulsions. There was no visible change in the emulsions by ultrasound. The particle size distribution of emulsions simultaneously transitioned from bimodal distribution to monomodal distribution, and the peak values of emulsions shifted to lower values with increased ultrasound times. The emulsions maintained uniform distribution and a smaller particle size by the microstructure. The ultrasound facilitated droplet breakup and formed smaller droplets, preventing droplet aggregation or coalescence after the freeze–thaw. In addition, the interface layer plays a significant role in the emulsions’ freeze–thaw stability by suppressing droplet coalescence. Ultrasound homogenization enhanced the emulsifying properties of emulsions discussed in Section 3.2 and implied that increasing the amount of protein adsorbed in the O/W interface formed a compact interfacial film, which improved steric repulsion between droplets highly resistant to coalescence. The reason for unstable emulsions by simple homogenization may be that the interfacial layers surrounding droplets were relatively thin. Oil droplets can be easily accessed and merged with each other. The results confirmed that emulsions by ultrasound had relatively fine freeze–thaw stability. 

**Figure 9:**
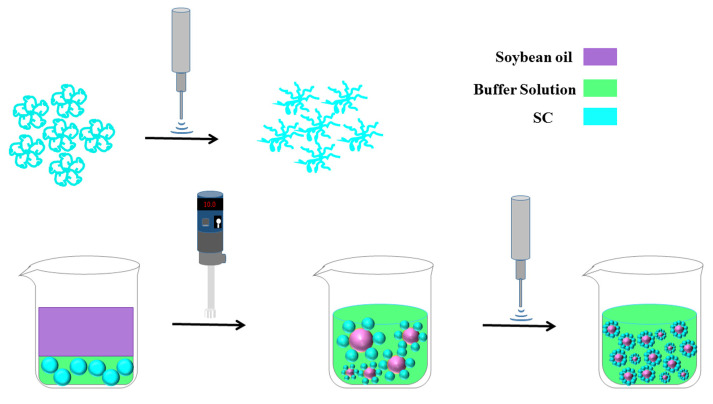
Proposed mechanism of the relation between the stability of SC-coated O/W emulsions and the physicochemical and structural characteristics of SC by a high-intensity ultrasound.

According to the above results, a proposed mechanism is shown in Figure 9. After the ultrasound treatment, SC had some physicochemical and structural changes, including reduced particle size, increased surface hydrophobicity and fluorescence intensity, exposure of tyrosine and tryptophan to the protein surface, and the formation of small aggregates and a loose structure. During the ultrasound homogenization process, more SC can be adsorbed to the interface of oil droplets, forming the stability of emulsions due to the above physicochemical and structural changes of SC. Under various environmental stresses, the stability of ultrasound-treated emulsions inhibited flocculation and coalescence. High-intensity ultrasounds enhanced SC-coated O/W emulsion stability.

Emulsified meat products, such as sausages, meatballs and burgers, are the most popular ready-to-eat foods for consumers. In general, emulsified meat products have a high level of fat, ranging from 20% to 30%. Excessive consumption threatens human health and easily leads to obesity and certain chronic diseases. The better method to replace animal fat in emulsified meat products is the application of pre-emulsification, that is, using non-meat proteins instead of animal proteins to wrap fat globular, and then using pre-emulsified vegetable oil instead of pig back-fat. However, the pre-emulsion prepared in the actual production process is unstable and needs to be processed into the minced meat system in time. The ultrasound treatment enhanced the SC-coated O/W emulsions stability, which could be applied in the industrial processing of emulsified meat products.

## 4. Conclusions

The results revealed that ultrasound treatment might destroy the tertiary structure but leave most of the integral secondary structure by reducing particle size, increasing surface hydrophobicity and fluorescence intensity, exposing tyrosine and tryptophan to the protein surface and forming small aggregates and a loose structure of SC. The physicochemical and structural changes were closely related to the enhanced SC adsorption to oil droplets and emulsions stability. SC stabled O/W emulsions by the ultrasounds exhibited excellent stability under environmental stresses. The oil droplets of treated emulsions after storage and the thermal and freeze–thawing process were still uniform and stable, especially for 6 min and 9 min. The stabilization mechanism for ultrasound-induced emulsion prepared by SC as an emulsifier needs further study. The high-intensity ultrasounds have the potential to enhance the stability of protein-prepared O/W emulsions and are of great significance in practical emulsified products.

## Figures and Tables

**Table 1:** Effects of the high-intensity ultrasound treatment on the particle size and zeta potential of SC.

Ultrasonic Time (min)	Particle Size (nm)	Zeta Potential (mV)
0	274.9 ± 5.1 ^a^	−11.73 ± 1.01 ^a^
3	241.0 ± 3.7 ^b^	−13.07 ± 0.32 ^a^
6	208.3 ± 3.2 ^c^	−12.98 ± 0.50 ^a^
9	205.1 ± 9.1 ^c^	−12.76 ± 0.83 ^a^

^a–c^: Different letters above standard deviation bars are significantly different (*p* < 0.05).

**Table 2:** Effect of high-intensity ultrasound treatment on the secondary structure of SC.

Ultrasonic Time (min)	α-Helix	β-Sheet	β-Turn	Random Coil
0	41.21 ± 1.42 ^a^	24.12 ± 0.77 ^a^	17.38 ± 0.64 ^a^	18.72 ± 0.51 ^a^
3	39.07 ± 2.89 ^a^	25.20 ± 0.40 ^a^	17.71 ± 0.72 ^a^	19.25 ± 1.52 ^a^
6	41.86 ± 1.05 ^a^	24.34 ± 0.71 ^a^	17.63 ± 0.79 ^a^	20.56 ± 1.23 ^a^
9	40.85 ± 2.45 ^a^	24.32 ± 0.05 ^a^	18.57 ± 0.06 ^a^	19.65 ± 1.35 ^a^

^a^: Different letters above standard deviation bars are significantly different (*p* < 0.05).

## Data Availability

The data presented in this study are available on request from the corresponding author.
